# Equine Veterinarian Perspectives on Mucocutaneous Tumors in Horses: A Survey-Based Study in Portugal

**DOI:** 10.3390/ani15131853

**Published:** 2025-06-23

**Authors:** José Pimenta, Mário Cotovio

**Affiliations:** 1 CIVG—Vasco da Gama Research Center, EUVG—Vasco da Gama University School, 3020-210 Coimbra, Portugal; 2 CECAV—Veterinary and Animals Research Center, University of Trás-os-Montes e Alto Douro, 5000-801 Vila Real, Portugal; p4980@ulusofona.pt; 3 Associate Laboratory for Animal and Veterinary Sciences (AL4AnimalS), 5000-801 Vila Real, Portugal; 4 Faculty of Veterinary Medicine, Lusófona University, Campo Grande 376, 1749-024 Lisbon, Portugal

**Keywords:** equine, oncology, survey, diagnostic, treatment, mucocutaneous, tumors

## Abstract

This study explores equine veterinarians’ clinical practices regarding mucocutaneous tumors, based on a survey conducted among members of the Portuguese Association of Equine Veterinarians, and examines their perceptions of owner and buyer concerns regarding these tumors. Veterinarians expressed significantly greater concern about squamous cell carcinoma (SCC) during routine examinations (*p* < 0.001) compared to sarcoids and melanomas. Concern over sarcoids was significantly greater during pre-purchase evaluations compared to routine clinical exams (*p* = 0.03). For melanomas, a trend toward increased concern in pre-purchase evaluations compared to routine examinations was observed (*p* = 0.07). There was a statistically significant association between the use of histopathology and the type of tumor (*p* < 0.001). Histopathology was performed less frequently in cases of melanoma compared to other tumor types. The cost was the main reason for not performing histopathology. During pre-purchase exams, buyers appeared to show greater concern regarding the presence of sarcoids (*p* = 0.001) and melanomas (*p* = 0.002) than regular owners. This study highlights the need to bolster training and education in equine oncology for veterinarians and owners to promote better diagnostic and therapeutic practices.

## Simple Summary

Through a survey, this study analyzes how Portuguese equine veterinarians diagnose and treat common mucocutaneous tumors and their perceptions on owner and buyer concerns. Veterinarians showed greater concern about squamous cell carcinoma (SCC) during routine exams compared to sarcoids and melanomas. Sarcoids were of greater concern during pre-purchase evaluations compared to routine clinical exams, and a trend toward increased concern for melanomas was observed during pre-purchase evaluations compared to routine exams. A significant association was found between the use of histopathology and tumor type, with fewer histopathological exams performed on melanomas. The primary reason for not performing histopathology was cost. Buyers showed greater concern regarding the presence of sarcoids and melanomas during pre-purchase evaluations compared to regular owners. This study identifies several gaps in clinical practice related to equine oncology, as well as in the knowledge and awareness of horse owners. It highlights the need for improved training for veterinarians and greater awareness among owners regarding the diagnosis and management of equine tumors.

## 1. Introduction

Equine oncology is a field of veterinary medicine that remains underdeveloped compared to other species [1,2,3,4,5,6]. Although oncology has not traditionally been considered as a central issue in equine medicine, its importance has been steadily growing [7,8]. One major driver for this is the growing population of geriatric horses, where the prevalence of these diseases is higher. Another possible justification is based on raised expectations among owners regarding treatments for equine neoplasms which mainly come from the noticeable advances they see in human oncology [9,10,11,12,13]. As a result, the demand for more innovative and evidence-based treatment options has never been higher. However, despite the growing interest and recognition of equine oncology, this area of equine medicine still faces significant challenges, especially regarding the relatively low incidence of neoplasia in horses, which directly impacts the amount of knowledge and experience available, hindering progress in both diagnosis and treatment [8,14,15,16,17,18].

Among the most diagnosed neoplasms in horses are mucocutaneous tumors, including sarcoids, squamous cell carcinoma (SCC), and melanomas [18,19,20]. Sarcoids are often locally aggressive, with a tendency to recur even after multiple treatments. This recurrence makes them difficult to manage, especially when they appear in challenging anatomical locations [18,21,22]. SCC, while less common, presents much more aggressive and malignant behavior, leading to a greater risk of metastasis, often requiring invasive treatments [20,23]. Melanomas typically affect older horses with grey coat and can present a broad spectrum of clinical behaviors, ranging from relatively benign forms to more aggressive and life-threatening conditions [24,25].

The overall deficit of research in the field of equine oncology has resulted in a lack of high-quality literature providing evidence-based clinical approaches and comprehensive guidelines for most tumor types. Therefore, there is considerable variability in how different clinicians approach the diagnosis and treatment of equine mucocutaneous neoplasia’s [26,27,28,29]. Many of these approaches are often based on case reports, empirical knowledge, or personal experience rather than solid, evidence-based practices [8,10,29,30]. This variability in clinical approaches hampers treatment success and the standardization of treatment protocols

Field veterinarians, who are often the first point of contact for horse owners, have a significant role in identifying, diagnosing, and managing neoplastic conditions. Their ability to recognize early signs of neoplasia and make informed decisions on treatment options has direct implications on the overall prognosis of the affected horses [31]. Understanding how these professionals approach these tumors, both in terms of diagnosis and treatment and their degree of concern about each type of mucocutaneous tumor, can bring valuable insights. By understanding how they diagnose, treat, and communicate about tumors, especially those of the mucocutaneous which are the most common, researchers and practitioners can work together to identify gaps in knowledge and improve clinical practices, as has been achieved and published for other diseases [32,33,34,35]. Furthermore, understanding veterinarians’ perceptions of horse owners’ knowledge and concern about mucocutaneous tumors, as well as the impact that these tumors can have, for example, during a pre-purchase examination, can help veterinarians address owners properly and guide informed decisions [36,37,38]. This insight is vital in fostering better client–veterinarian relationships and ensuring more effective management of equine mucocutaneous tumors.

By surveying Portuguese equine veterinarians, this study aims to assess their diagnostic and therapeutic approaches to the most common mucocutaneous tumors, as well as examine their perceptions of the concerns and expectations of horse owners/buyers when it comes to dealing with these neoplasia’s, not only during routine clinical examinations, but also including the impact in the pre-purchase examinations.

To the best of the authors’ knowledge, no similar scientific study has been published to date. This is the first study that is aiming to build an understanding of veterinarians’ perceptions and approaches regarding various aspects of equine mucocutaneous tumors through a survey.

## 2. Materials and Methods

### 2.1. Survey Design

An online survey was designed using the Google Forms platform and distributed via email and social media platforms to all members of the Portuguese Association of Equine Veterinarians, being available between November of 2024 and March of 2025.

Before the announcement, the questionary was pilot-tested for user-friendliness by some invited participants (n = 10). According to external comments and suggestions, the survey was adapted; however, these data were not included in the results of this work. The survey received ethical approval from the Ethics Committee of the Vasco da Gama University School (N° 30/2024). Before starting the survey, participants were provided with all the information about it, acknowledging that the questionary was anonymous and that data would be used for scientific purposes. All participants gave their informed consent prior to beginning the survey. Furthermore, participants had the option to withdraw at any point during the survey.

The survey was divided into three sections: (1) veterinarians profile; (2) experience with mucocutaneous tumors; (3) veterinarians’ opinions on owners’ awareness about mucocutaneous tumors.

In the first section of the survey, the objective was to characterize the sample in terms of gender, professional experience, academic degree, type of clinical practice, geographical area of intervention, as well as breed and discipline/use of the horses they work with.

In the second part, the questions aimed to understand the case load regarding mucocutaneous tumors, the most common tumors in their clinical practice, their level of concern (1—very low; 2—low; 3—moderate; 4—high; 5—very high) when diagnosing a mucocutaneous tumor during clinical check-up or during a pre-purchase examination, and understand their diagnostic and therapeutic approach to these tumors. To ensure objective responses, the questions focused on the most common equine mucocutaneous tumors, namely sarcoids, SCC, and melanomas.

In the third section of the survey, the questions aimed to understand veterinarians’ opinions regarding owners/buyers’ level of concern when a mucocutaneous tumor is diagnosed during a routine appointment requested for an unrelated primary reason or a pre-purchase examination and owner’s willingness to pursue treatment for each of these tumors.

The survey consisted of multiple-choice questions, and, in some cases, the participants had the opportunity to write in an open comment box. These open boxes allowed the participant to provide further explanation for their response or to provide an answer not comprised in the multiple-choice options.

EPITOOLS epidemiological calculator was used to estimate the minimum necessary sample size (minimum number of responses) considering that the total population of active members of the Portuguese Association of Equine Veterinarians was 131 members. Based on the reported very low rate of responses to online surveys, especially in equine medicine, a 90% confidence interval and a 10% margin error were applied to this work as suggested by [39,40,41]. A minimum of 45 responses/participants were required, considering the total population (n = 131).

The full questionnaire is available in the Supplementary Materials S1.

### 2.2. Data Analysis

Data were collected directly from Google forms into Microsoft Excel and checked for inconsistencies in responses.

Descriptive statistics were made to explore relative frequencies (%) for most data. Shapiro–Wilk test was performed to assess the normal distribution of data. For inferential statistics, Chi-square and Fisher tests were used for categorical variables and non-parametric tests Mann–Whitney and Kruskal–Wallis were performed for ordinal or continuous variables. Results were considered significant when *p* < 0.05. Statistics were performed using Jamovi 2.3.28.

## 3. Results

This survey included 61 participants, which corresponds to 47% of the target population.

### 3.1. Veterinarians Profile

Of the 61 participants, 52.5% (n = 32) were female and 47.5% (n = 29) were male.

Many participants (77%) held a Master’s degree in Veterinary Medicine, while 11.5% had a PhD, and 11.5% held a pre-Bologna Bachelor’s degree. Most respondents (70.5%) practiced exclusively equine medicine, whereas the remaining 29.5% worked in mixed practice: equine and livestock (13.1%); equine and small animals (13.1%); or equine, livestock, and small animals (3.3%).

Regarding clinical experience, based on years of practice, 42.6% of participants had more than 10 years of experience, 11.5% had more than 6 years, and 45.9% had fewer than 5 years.

In terms of clinical areas of expertise, participants were allowed to select multiple fields. General equine practice (67.2%, n = 42) and sports medicine (45.9%, n = 28) were the most frequently reported areas.

Concerning the type of practice, 60.7% of veterinarians worked in ambulatory practice, 21.3% in hospital-based practice, and 18% in both settings.

Geographically, 77.1% of the participants worked in mainland Portugal, distributed across North Portugal (24.5%), Central Portugal (29.5%), and South Portugal (23%) regions, while 23% also practiced outside the country.

The veterinarians’ caseload predominantly involved Lusitano Purebred, Warmblood, or crossbred horses, with dressage, leisure riding, and show jumping being the most common disciplines among the horses they treated.

### 3.2. Experience with Mucocutaneous Tumors

**Figure 1:**
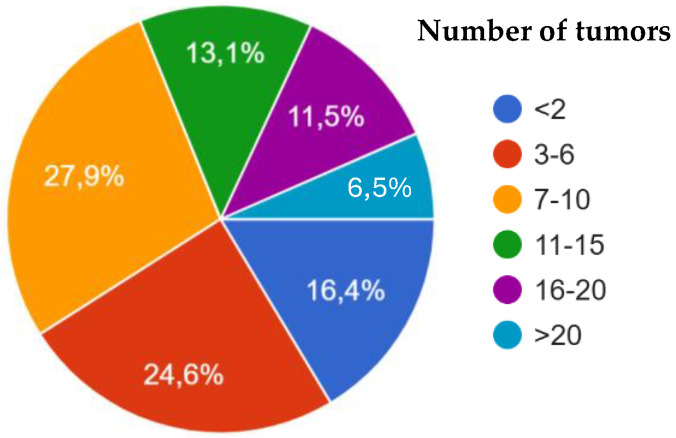
Distribution of the caseload of mucocutaneous tumors per year.

Regarding general case distribution, 68.8% of veterinarians encountered fewer than 10 cases of equine mucocutaneous tumors per year, while 31.1% reported managing more than 10 cases annually (Figure 1).

**Figure 2:**
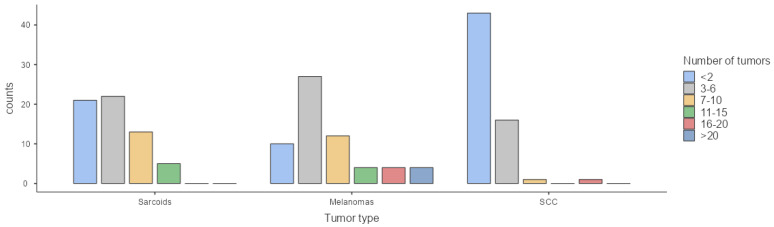
Caseload of each tumor type per year.

For sarcoids, 70.5% of respondents managed fewer than six cases annually, whereas 29.5% reported seeing more than seven cases per year. In the case of melanomas, 16.4% of veterinarians treated less than 2 cases per year, 44.3% managed 3–6 cases annually, 19.7% reported 7–10 cases, and 19.8% of veterinarians treated more than 10 cases per year. SCC appeared to be less frequently diagnosed, with 70% of veterinarians reporting fewer than 2 cases per year, 26.6% managing 3–6 cases, 1.7% seeing 7–10 cases, and 1.7% encountering 16–20 cases per year (Figure 2). The annual number of sarcoid and melanoma cases was significantly higher than that of SCC (*p* < 0.001), although no significant difference was found between sarcoid and melanoma case numbers (*p* = 0.059). No association was detected between the clinical practice setting (hospital-based vs. ambulatory) and the case frequency of sarcoids (*p* = 0.95), melanomas (*p* = 0.27), or SCC (*p* = 0.38). No association between geographical area of intervention and caseload of tumors was found either (*p* = 0.4).

Additionally, 14.8% of participants reported having diagnosed cutaneous lymphoma, and 4.9% had identified mast cell tumors.

#### 3.2.1. Clinical Concern on Mucocutaneous Tumors

The level of clinical concern during a routine examination requested for an unrelated primary reason was assessed for each of the three most common types of equine mucocutaneous tumors.

**Figure 3:**
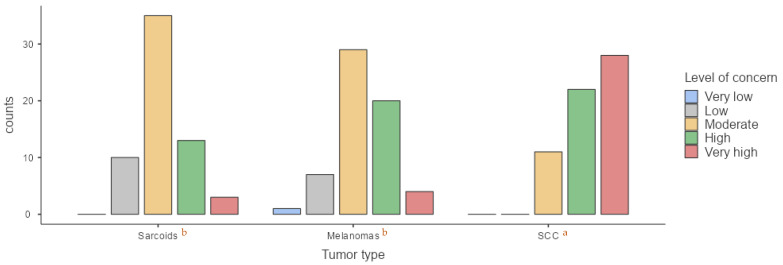
Comparison of veterinarian’s level of concern during routine clinical examination regarding each type of tumor. (a) According to Kruskal–Wallis test, significant differences were observed in the medians between SCC and melanomas and sarcoids (*p* < 0.001); (b) no significant differences were observed in the medians of level of concern between sarcoids and melanomas (*p* = 0.4).

For sarcoids, 16.4% of participants reported a low level of concern, 57.4% a moderate level, 21.3% a high level, and only 4.9% a very high level. When evaluating melanomas, 1.6% of veterinarians expressed very low concern, with 11.5% expressing low concern, 47.5% expressing moderate concern, 32.8% expressing high concern, and 6.6% expressing very high concern. Regarding SCC, 18% of respondents reported a moderate level of concern, 36.1% reported high concern, and 45.9% expressing very high concern (Figure 3). Statistically significant differences were observed in level of concern during routine examinations depending on tumor type. SCC raised significantly greater concern (*p* < 0.001) compared to sarcoids and melanomas, although no significant difference was found between the latter two (*p* = 0.4).

**Figure 4:**
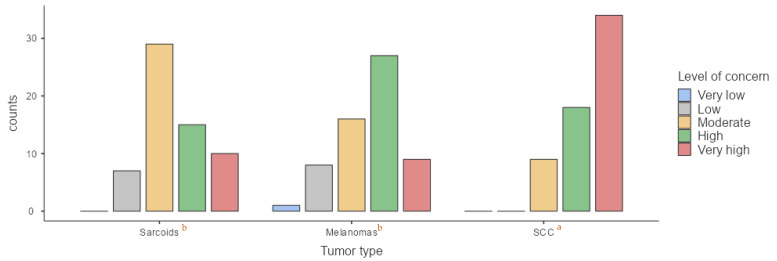
Comparison of veterinarian’s level of concern regarding each type of tumor during pre-purchase exam. (a) According to Kruskal–Wallis test, significant differences in the medians were observed between SCC and melanomas (*p* < 0.001) and between SCC and sarcoids (*p* < 0.001); (b) no significant differences were observed between sarcoids and melanomas (*p* = 0.21).

To assess potential changes in perception depending on the context, we also evaluated reactions to these tumors during a pre-purchase examination. In this scenario, 11.5% of respondents reported a low level of concern for sarcoids, 47.5% expressed moderate concern, 24.6% expressed high concern, and 16.4% expressed very high concern. Concern over sarcoids was significantly greater in pre-purchase evaluations compared to routine clinical examinations (*p* = 0.03). For melanomas, 1.6% of respondents reported very low concern during a pre-purchase examination, 26.2% expressed moderate concern, 44.3% expressed high concern, and 14.8% expressed very high concern. A trend toward increased concern in pre-purchase evaluations compared to routine examinations was observed (*p* = 0.07), although it did not reach statistical significance. For SCC, 14.8% of participants reported moderate concern, 29.5% expressed high concern, and 55.7% expressed very high concern (Figure 4). No significant differences were found between routine and pre-purchase examinations regarding concern levels for this tumor type (*p* = 0.31). Furthermore, a statistically significant difference was observed in concern levels during pre-purchase examinations depending on tumor type. Concern was significantly higher for SCC (*p* < 0.001) compared to sarcoids and melanomas, as previously noted for routine examinations.

No association was found between the level of concern and years of experience or type of clinical practice (exclusively equine medicine or mixed practice) for any of the mucocutaneous tumor types.

#### 3.2.2. Diagnostic Approach to Mucocutaneous Tumors

Regarding the diagnostic approach, the objective was to assess whether equine veterinarians routinely recommend/perform histopathological analysis when encountering a suspected mucocutaneous tumor, as recommended in the literature, or if they base their diagnosis solely on clinical examination.

Table 1 summarizes the decisions regarding histopathological submission for each type of tumor. A statistically significant association was observed between the performance of histopathology and tumor type (*p* < 0.001). SCC is the tumor type most frequently subjected to histopathological analysis by respondents compared with sarcoids (*p* = 0.035) and melanomas (*p* < 0.001). Although histopathology was performed less frequently for melanomas, there was no significant difference between melanomas and sarcoids in this regard (*p* = 0.103)

Clinicians who reported not performing histopathological analyses were given the opportunity to explain or justify their decision, with participants being able to provide multiple reasons. Regarding sarcoids, 15 veterinarians reported not submitting the tumor for histopathological analysis. Considering that each participant could mention multiple justifications, a total of 16 justifications were provided. The most common reason was that owners were unwilling to pay for histopathological analysis (7/16, 43.8%); followed by the fact that clinicians based their diagnosis on clinical examination of the lesion, not requiring analysis (6/16, 37.5%); the belief that sarcoids are benign tumors with minimal impact on the horse’s health, thus not requiring histopathological examination (2/16, 12.5%); and finally, the use of therapies other than surgery, which did not provide a sample for submission (1/16, 6.3%). Regarding melanomas, and considering that each participant could mention multiple justifications, a total of 35 reasons were recorded from the 26 clinicians who reported not performing histopathological analysis on these tumors. The following reasons were reported: clinicians relying on the clinical evaluation of the lesion for diagnosis (18/35, 51.4%); owners being unwilling to pay for histopathological analysis (13/35, 37.1%); and some clinicians considering melanoma to be a benign tumor with minimal consequences for the horse, thus not requiring histopathological examination (4/35, 11.4%). Among the few participants who reported not performing histopathological analysis of SCC (n = 8), five (62.5%) mentioned that owners were unwilling to pay, while three (37.5%) stated that they relied on clinical examination for diagnosis, considering histopathological analysis unnecessary.

The decision to perform histopathological analysis did not vary with years of clinical experience (sarcoids *p* = 0.25; melanomas *p* = 0.76; SCC *p* = 0.74).

#### 3.2.3. Therapeutical and Preventive Approach to Mucocutaneous Tumors

Regarding the therapeutic approach, one of the primary objectives was to assess whether clinicians alert owners and recommend treatment when they detect a tumor during a clinical examination conducted for an unrelated primary reason. Additionally, we assessed the most commonly used treatments, the timing chosen for intervention, and whether clinicians have ever had the need to refer cases of mucocutaneous tumors to more specialized treatment.

Data regarding participants’ responses on whether they alert owners to the presence of a tumor and recommend treatment are presented in Table 2. There was a statistically significant association (*p* = 0.001) between the type of tumor and the clinicians’ attitude towards detecting a tumor during a clinical examination; most of them highlight the issue and recommend treatment for SCC, while melanoma was apparently the most negligible.

Reasons for not alerting/recommending treatment in sarcoids included the following: the owner did not ask for the tumor to be examined; sarcoids may become more aggressive after intervention. Veterinarians also mentioned that the decision depends on the type of sarcoid, with some types prompting alerting/recommending treatment and others not.

Reasons for not alerting/recommending treatment of melanomas included: owners do not show interest in treating melanomas, the idea that melanomas do not progress clinically, and the belief that melanomas may become more aggressive after treatment.

Regarding the ideal timing for intervention, the data are presented on Table 3. The recommended timing for intervention varied significantly by tumor type (*p* = 0.004), with SCC being treated the earliest, while melanoma tends to be addressed later.

In an open comment box, some participants mentioned the reasons for advising late intervention. In the case of sarcoids, the type of sarcoid, the possibility that intervention could increase the tumor’s aggressiveness, and the requisition for an exam by a different reason are factors that lead the clinicians to either not recommend intervention or to delay it. For melanomas, the lack of owner interest in treating these tumors, the benign nature and slow progression of these tumors, and the possibility that intervention could make them more aggressive were reported as factors that lead clinicians to delay intervention.

Regardless of the timing recommended by veterinarians for melanoma intervention, 72% reported that owners only seek evaluation and treatment once the melanoma is already long-standing, has grown significantly, and has become a concern for the owners. Additionally, 16.5% reported being called only when melanomas ulcerate, while only 11.5% stated that they are contacted to treat small melanomas that have emerged recently.

To assess which treatments are most frequently used for each type of tumor, participants could select or write down the various treatment options they work with. For sarcoids, classical surgery was the most commonly performed treatment (44/61, 72%), followed by cryosurgery (23/61, 38%), laser or electrocautery (20/61, 33%), elastrator (14/61, 23%), surgery with adjuvant intratumoral chemotherapy (12/61, 20%), AW5 (9/61, 15%), immunotherapy with Bacillus Calmette–Guérin vaccine (9/61, 15%), imiquimod (7/61, 11%), acyclovir (5/61, 8%), bloodroot (XXTERRA^®^ Larson Laboratories DBA Vetline Equine, Inc., Fort Collins, CO, USA) (3/61, 5%), and tigilanol tiglate (1/61, 2%). In the case of melanomas, classical surgery was also the most used treatment (48/61, 77%), followed by cimetidine (24/61, 39%), laser or electrocautery (15/61, 25%), Oncept^®^ vaccine (6/61, 10%), cryosurgery (4/61, 7%), surgery with adjuvant intratumoral chemotherapy (4/61, 7%), and intratumoral chemotherapy only (2/61, 3%). Finally, for SCC, classical surgery remained the most used treatment (29/61, 48%). However, unlike the other tumors, some participants referred the case immediately (10/61, 16%). Laser or electrocautery (9/61, 15%), surgery with adjuvant intratumoral chemotherapy (5/61, 8%), cryosurgery (4/61, 7%), intratumoral chemotherapy alone (2/61, 3%), COX-2 inhibitors (1/61, 2%), and 5-fluorouracil (1/61, 2%) were also reported.

Veterinarians were specifically asked whether they had achieved good results in melanoma cases using the Oncept^®^ vaccine (Boehringer Ingelheim Animal Health USA Inc., Duluth, GA, USA). A total of 4.9% reported successful outcomes, 4.9% reported no success, and 90.2% stated that they had never used it.

The same question was posed regarding oral cimetidine, with 21.3% reporting good results, 18% reporting no success, and 60.7% stating they had never used it.

When asked if it had ever been necessary to refer cases for specialized treatment, the answers varied slightly according to tumor type, as illustrated on Table 4. Although there seems to be a greater tendency to refer melanomas, no association was observed between the type of tumor and the referral of cases (*p* = 0.13).

In an open response box, it was possible to write the reasons for referring the cases. The reasons were the same for the three tumor types: size of the tumor, location of difficult surgical access, need for chemotherapy or need for advanced surgical technique (tail amputation or phallectomy).

In terms of a preventive approach, 78.7% of clinicians believe that is not possible to prevent sarcoid and 21.3% believe it is possible. Among the measures indicated for prevention are the management of flies and other insects, avoiding surgical procedures in the field during the summer, and proper wound management. Of the participants, 85.2% do not think it is possible to prevent melanomas, 14.8% think it is possible with the Oncept^®^ vaccine or by avoiding the use of horses homozygous for the grey gene for breeding. Of the participants, 88.5% did not consider SCC prevention to be feasible, while 11.5% said that protection against UV radiation could be a form of prevention.

### 3.3. Veterinarians’ Opinion on Owners’ Awareness About Mucocutaneous Tumors

Veterinarians’ perception of owners’ concern upon being informed, during a routine clinical examination requested for an unrelated primary reason, that their horse has a mucocutaneous tumor was assessed.

**Figure 5:**
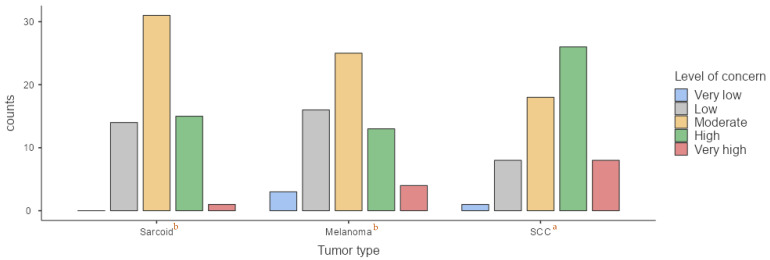
Comparison of owner’s level of concern regarding each type of tumor during routine clinical examination. (a) According to Kruskal–Wallis test, significant differences in the medians were observed between SCC and sarcoids (*p* = 0.012) and melanomas (*p* = 0.001); (b) no significant differences were observed between sarcoids and melanomas (*p* = 0.83).

Of the veterinarians, 23% reported that owners exhibit low level of concern regarding sarcoids; 50.8% reported a moderate level of concern; 24.6% reported a high level of concern, and 1.6% reported a very high level of concern. Regarding melanomas, 4.9% reported a very low level of concern; 26.2% reported a low level of concern; 41% reported a moderate level of concern; 21.3% reported a high level of concern; 6.6% reported a very high level of concern. For SCC, 1.6% of veterinarians perceived that owners exhibit a very low level of concern; 13.1% reported a low level of concern; 29.5% reported a moderate level of concern; 42.6% reported a high level of concern; 13.1% reported a very high level of concern. Statistically significant differences were detected in owners’ levels of concern depending on tumor type (Figure 5). Concern was significantly higher for SCC compared to sarcoids and melanomas (*p* = 0.012; *p* = 0.001, respectively), but no significant difference was found between sarcoids and melanomas (*p* = 0.83).

When comparing the levels of concern regarding the presence of a sarcoid during a routine examination between veterinarians and owners, no significant differences were observed (*p* = 0.64). However, veterinarians showed a significantly higher level of concern compared to owners regarding melanomas (*p* = 0.04) and SCC (*p* < 0.001).

**Figure 6:**
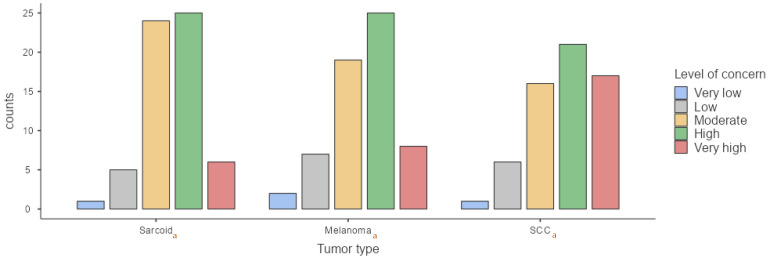
Comparison of buyer’s level of concern regarding each type of tumor during pre-purchase examination. (a) According to Kruskal–Wallis test, no significant differences were observed in the medians between sarcoids and melanomas (*p* = 0.98); SCC and sarcoids (*p* = 0.18) or SCC and melanomas (*p* = 0.27).

To assess the impact of mucocutaneous tumors on pre-purchase examinations, veterinarians’ perceptions of buyers’ concern upon identifying a mucocutaneous tumor during a pre-purchase evaluation were analyzed. According to veterinarians’ perceptions, 1.6% reported that buyers exhibit a very low level of concern regarding sarcoids; 8.2% reported that buyers exhibit a low level of concern; 39.3% reported that buyers exhibit a moderate level of concern; 41% reported that buyers exhibit a high level of concern; 9.8% reported that buyers exhibit a very high level of concern. For melanomas, 3.3% reported a very low level of concern; 11.5% reported a low level of concern; 31.1% reported a moderate level of concern; 41% reported a high level of concern; 13.1% reported a very high level of concern. Regarding SCC, 1.6% reported a very low level of concern; 9.8% low; 26.2% reported a moderate level of concern; 34.4% reported a high level of concern; 27.9% reported a very high level of concern (Figure 6). No significant differences were found in buyers’ levels of concern based on tumor type (*p* = 0.16).

When comparing the level of concern between buyers and regular owners, buyers appeared to show greater concern regarding the presence of sarcoids (*p* = 0.001) and melanomas (*p* = 0.002) than regular owners. However, no significant differences were found for SCC (*p* = 0.153).

When comparing the level of concern between veterinarians and buyers, no significant differences were found for sarcoids (*p* = 0.6) or melanomas (*p* = 0.64). However, veterinarians exhibited a significantly higher level of concern regarding SCC (*p* < 0.001).

According to Table 5, statistically significant differences were observed in owners’ willingness to invest in treatment depending on the type of tumor (*p* = 0.014). Motivation to treat sarcoids and SCC was significantly higher than for melanomas (*p* = 0.04 and *p* = 0.02, respectively), but no significant differences were found between sarcoids and SCC (*p* = 0.97).

## 4. Discussion

According to the reports from Portuguese equine veterinarians who participated in this study, sarcoids and melanomas are diagnosed significantly more often than SCC. This finding aligns with the existing literature, identifying sarcoids as the most prevalent mucocutaneous tumor in horses [18,42,43]. This study was carried out in Portugal, where most clinicians work with the Lusitano breed, comprising many grey horses; this may justify why melanoma was the second most common neoplasm, unlike other articles which consider SCC to be the second most common [20]. The case frequency of melanomas reported in this work (40% of respondents reported seeing more than seven melanomas per year) underscores the need for ongoing clinical vigilance and research efforts about new therapeutic options for this tumor. Although SCC appeared to be infrequent (70% of respondents reported diagnosing fewer than two cases per year), the concern with this tumor is high, as discussed below.

During routine clinical examinations, SCC elicited the highest level of clinical concern, consistent with its greater metastatic potential [16,23]. In contrast, sarcoids and melanomas were generally associated with moderate concern, with no significant difference between them. However, this survey did not distinguish between sarcoid subtypes, which vary in clinical behavior—ranging from the relatively indolent occult and verrucous forms to the more aggressive fibroblastic type [18,21,22]. This may have influenced the results, as more aggressive sarcoids may raise greater concern than melanomas, while less aggressive forms may be perceived as less concerning, potentially balancing the overall comparison. Additionally, although not extensively evaluated in this study, tumor location may influence the level of concern. For example, a melanoma located in the parotid region or eyelid may cause greater concern due to the potential complications and treatment challenges it presents. Similarly, a periocular sarcoid is likely to raise more immediate concern than a sarcoid located on the trunk.

When comparing concerns during routine clinical examinations requested for unrelated primary reason and pre-purchase exams, concern over sarcoids increased significantly, suggesting that veterinarians may place additional weight on the potential implications of these tumors when advising on the suitability or long-term soundness of a horse prior to sale. This heightened concern may stem from the unpredictable behavior and high recurrence rate of sarcoids, which can complicate future management. Moreover, the genetic component of sarcoid development (ELA-linked gene) implies that horses affected by this tumor have a higher likelihood of producing offspring that will also develop sarcoids [18,22,44,45,46]. This consideration is particularly relevant in animals with breeding potential, as it may influence decisions regarding their use in reproductive programs. Sarcoids are a chronic and recurrent condition that may negatively impact the horse’s long-term management, resale value, and suitability for certain activities [22,26,47].

Although the increase in concern over melanomas during pre-purchase evaluations did not reach statistical significance, a clear trend was observed. Melanoma has a significant global impact on the trade of genetically predisposed grey horses [42,48,49,50]. This impact has already been observed in the Lusitano breed, where a notable decline in the number of grey horses has been reported [51]. This reduction is attributed to selective breeding pressure applied by breeders, who aim to avoid the grey phenotype due to its association with melanoma. The presence of this tumor, its progression over time, and the potential need for surgical excision (often requiring advanced surgical techniques) may have crucial weight in pre-purchase examinations [52]. In this context, the potential for future progression and future complications of melanomas may weigh more heavily ding pre-purchase exams, even if the short-term clinical impact appears limited.

SCC maintained the highest level of concern across both clinical contexts, with no significant difference between routine and pre-purchase evaluations. The consistently elevated concern, irrespective of context, may indicate that veterinarians consider SCC to be an unequivocal clinical priority when detected.

Taken together, these results suggest that veterinarians modulate their level of concern based not only on the tumor type but also on the purpose of the examination. Tumor behavior, recurrence risk, management difficulties, and implications for resale appear to play key roles in shaping clinical judgment. These findings underscore the importance of context-specific communication with clients, particularly during pre-purchase evaluations, when the long-term consequences of mucocutaneous tumors may have greater implications for decision-making.

Our findings reveal that, while most veterinarians report performing histopathological analysis when presented with suspected mucocutaneous tumors, many do not. This reflects a complex interplay of clinical judgment, economic constraints, and tumor biology. The high compliance with subjecting suspicious SSC to histopathology likely reflects the tumor’s recognized malignant behavior, which highlights the need to assess some characteristics such as vascular and lymphatic invasion, mitotic count, and nuclear grade [53,54,55]. Additionally, the early stage and well-differentiated SCCs can be confused clinically with exuberant granulation tissue or *habronema* spp. lesions in the absence of histological evaluation. This can lead to inappropriate treatment of the tumor in question, which may have serious consequences for the animal’s life such as metastasis [20,56].

While the vast majority of sarcoids are submitted to analysis, the fact that nearly one-quarter of veterinarians forego histopathological confirmation is noteworthy. This may reflect the characteristic clinical appearance of many sarcoids, which can lead to overconfidence in presumptive diagnosis based on visual inspection alone. However, given the similarity of some types of sarcoids with other neoplastic and non-neoplastic lesions such as melanomas, fibromas, SCC, and granulation tissue, reliance on clinical diagnosis alone carries the risk of misclassification and subsequent mismanagement [57]. Furthermore, the concern that iatrogenic-induced trauma may trigger progression toward more aggressive forms could discourage clinicians from pursuing surgical excision/biopsy and, consequently, histopathological analysis [14,54,57,58]. Nevertheless, histopathology plays a crucial role by determining whether the sarcoid was excised with wide and clean margins, since incomplete excision significantly increases the likelihood of recurrence, which should be transmitted to the owner [14,26,55].

The lowest rate of histopathological confirmation was observed for melanomas, with 42.5% of respondents not submitting these tumors for analysis. This finding suggests a tendency among veterinarians to rely on clinical diagnosis, likely due to the typical anatomical location, pigmented appearance, and high prevalence in grey horses, which can make melanoma relatively straightforward to identify [42]. Moreover, the long-standing notion that all equine melanomas are benign may contribute to clinical underestimation and neglect [42,48,49,59]. In contrast to canine and human melanomas, histological features of malignancy in equine melanocytic tumors do not correlate well with clinical behavior. Tumors displaying histological markers of malignancy often exhibit indolent or non-aggressive clinical courses [5,59]. This incongruity may discourage clinicians from submitting samples for histopathological evaluation, as the histological output may be perceived as clinically irrelevant.

One of the most cited reasons for not submitting tumors for histopathological analysis—shared across all three tumor types—was the owner’s unwillingness to cover the associated costs. Our findings are consistent with reports in the literature, which highlight how economic constraints frequently influence clinical decision making and may lead to suboptimal diagnostic accuracy and therapeutic outcomes [60].

One of the reasons for not alerting owners to the presence of a sarcoid or for delaying intervention on these tumors was that “the owner did not ask for the tumor to be examined.” In the authors’ view, this approach cannot be considered the ideal clinical practice. Regardless of the primary reason for the examination, the horse should be assessed holistically, and all clinical findings should be communicated to the owner [8,31,37].

The reasons for not alerting owners or recommending treatment for melanomas often stem from misconceptions or owner reluctance. Many veterinarians cite the belief that melanomas do not progress clinically over time, which was already scientifically refuted [52]. Additionally, some veterinarians expressed concerns that treatment may induce melanoma aggression, although there is no evidence to support this either [61,62,63]. Nevertheless, owner disinterest in pursuing treatment is, unfortunately, a common reality, as observed during the author’s clinical experience. This mindset contributes massively to delayed intervention, which is also reflected in these results since 31.1% of the veterinarians recommend late intervention, and most participants reported that owners only request intervention when tumors have reached a considerable size or are already ulcerated and causing significant clinical impacts.

Regarding treatment, the most frequently used therapies reported for the three types of tumors were indeed those most described in the literature, particularly surgical excision [20,23,26,30]. The widespread use of classical surgery may also be influenced by the fact that many participants work in ambulatory practice, where traditional surgical tools are more readily available. However, this type of therapy is more successful and easier to apply in small tumors located in easily accessible locations. When the tumors are larger and in complicated locations, other types of therapy may be necessary, or referral to more experienced surgeons [28].

The Oncept^®^ vaccine for equine melanoma appears to have fallen into disuse as a therapeutic option. The main reason for its declining use may be the discrepancy between the initial promise and the actual clinical outcomes [25,64,65,66,67]. It appears that it only delays the progression of existing melanomas, with reduction in tumor volume being unlikely [25,66]. In contrast, cimetidine appears to remain in use by a larger number of clinicians, although clinical outcomes remain inconsistent. Given that cimetidine exerts its effects through immune system activation, future clinical studies should incorporate molecular characterization of the tumors to better identify responsive subtypes [17,68,69,70].

When assessing therapeutical approach to SCC, the heightened concern is underlined, since 16% of clinicians opt for immediate referral. The higher success rate associated with laser surgery combined with chemotherapy, which is more accessible in a hospital setting, may account for this approach [71,72,73].

In the literature, preventive measures are described for certain tumor types, and a few participants in this study appear to advise them accordingly [18,20,21,23,26,29,56]. However, given the very low percentage of clinicians who reported advising these measures (21.3% for sarcoids and 11.5% for SCC), there appears to be a need for greater education and awareness regarding these preventive approaches.

As expected, many respondents stated that melanoma cannot be prevented, which is consistent with the current literature. Strictly speaking, reduction in the use of homozygous grey horses does not constitute disease prevention, contrary to what was reported by the participants. Additionally, some clinicians incorrectly identified the use of the Oncept^®^ vaccine as a preventive measure. It is important to clarify that Oncept^®^ is a therapeutic, not a preventive vaccine, as it is intended for the treatment of existing tumors and does not prevent their development [64,65].

While veterinarians’ overall concern about melanomas is not as high as it could be, the level of concern among owners is significantly lower. Owners are generally more willing to allocate resources toward treating sarcoids and SCC, whereas in the case of melanomas, they are significantly less inclined to invest. However, all this perspective changes considerably when the monetary value of the horse is at stake. For a buyer, the potential impact of a mucocutaneous tumor, especially a sarcoid or melanoma, is much greater than for a regular owner. The buyer’s perspective is longer term, which places greater emphasis on these conditions. As so, veterinarians should be prepared to face different perspectives and mentalities for the same tumor type.

This study has some limitations. The fact that the questionnaire was conducted exclusively with Portuguese veterinarians does not allow the results to be extrapolated to clinicians in other countries, who may adopt different approaches. Additionally, only veterinarians registered with the Portuguese Association of Equine Veterinarians were included. As a result, some Portuguese veterinarians who work with horses but are not members of the association may have been inadvertently excluded. However, as a pioneering study, it paves the way for similar research to be conducted internationally, enabling a broader understanding of clinical practices related to equine mucocutaneous tumors. The sample size is also a limitation that may negatively impact the results. It should be noted that the survey did not consider veterinarians’ perceptions of owners who specifically called for a consultation because of a nodule or suspected tumor, and thus the results of this work may not reflect the general attitude of all owners towards mucocutaneous tumors.

## 5. Conclusions

This study provides insight into the perspectives and approaches of Portuguese equine veterinarians regarding mucocutaneous tumors. It clearly highlights that veterinarians’ level of concern varied notably based on the type of tumor and the clinical context. Squamous cell carcinoma raised the most concern both during routine check-ups and pre-purchase examinations, while sarcoids and melanomas prompted more awareness only in pre-purchase evaluations. Buyers tended to be significantly more worried about the presence of sarcoids and melanomas than regular owners. Diagnostic strategies also differed according to tumor type, with histopathology being used less often for melanomas. These results underscore the importance of enhancing veterinarians and owner education in equine oncology to promote greater knowledge, better diagnostic approach and greater awareness of the clinical relevance of mucocutaneous tumors in horses.

## Figures and Tables

**Table 1:** Relationship between tumor type and histopathological analysis.

	Histopathological Analysis			
Tumor Type	Yes	No	Total	*p*
**Sarcoid**	46 75.4%	15 24.6%	61	
**Melanoma**	35 57.4%	26 42.6%	61	**0.001**
**SCC**	56 91.8%	5 8.2%	61	
**Total**	137	46	183	

**Table 2:** The influence of tumor type on clinicians’ decisions to inform owners and suggest.

	Owner Alert and Treatment Recommendation			
Tumor Type	Yes	No	Total	*p*
**Sarcoid**	57 93.4%	4 6.6%	61	
**Melanoma**	48 78.7%	13 21.3%	61	**0.001**
**SCC**	61 100%	0 0%	61	
**Total**	166	17	183	

**Table 3:** Impact of tumor type on the timing for intervention recommended by clinicians.

	Timing for Intervention			
Tumor Type	Early	Late	Total	*p*
**Sarcoid**	52 85.2%	9 14.8%	61	
**Melanoma**	42 68.9%	19 31.1%	61	**0.004**
**SCC**	61 100%	0 0%	61	
**Total**	166	17	183	

**Table 4:** Relation between tumor type and referral for specialized treatment.

	Case Referral			
Tumor Type	Yes	No	Total	*p*
**Sarcoid**	19 31.1%	42 68.9%	61	
**Melanoma**	28 45.9%	33 54.1%	61	0.13
**SCC**	18 29.5%	43 70.5%	61	
**Total**	65	118	183	

**Table 5:** Owner willingness to invest in treatment according to tumor type.

	Owner Willingness to Invest in Treatment			
Tumor Type	Yes	No	Total	*p*
**Sarcoid**	51 83.6%	10 16.4%	61	
**Melanoma**	38 62.3	23 37.7%	61	**0.014**
**SCC**	50 82%	11 18%	61	
**Total**	138	45	183	

## Data Availability

Data are contained within the article.

## Supplementary Materials

The following supporting information can be downloaded at: https://www.mdpi.com/article/10.3390/ani15131853/s1. Supplementary Materials S1: full questionnaire.
