# Supplementary material for: Equine Veterinarian Perspectives on Mucocutaneous Tumors in Horses: A Survey-Based Study in Portugal
Source: Animals (Basel). 2025 Jun 23;15(13):1853. doi: 10.3390/ani15131853 (PMC12248879; doi:10.3390/ani15131853)
Supplement: Supplementary file 1 [file animals-15-01853-s001.zip › animals-3665665-supplementary.pdf]

Equine Veterinarians Perspective on Horse’s Mucocutaneous Tumors: a Survey-Based Study in Portugal

This survey is aimed at Portuguese veterinarians who work with horses, with the aim of evaluating the most commonly used diagnostic and therapeutic practices for mucocutaneous tumors.

This questionnaire is divided into 3 sections: “Veterinarians' profile”; “Experience with skin tumors”, “Veterinarians' opinion on owners awareness about mucocutaneous tumors".

The questionnaire is made up of several multiple-choice and short-answer questions and will take around 10 minutes to complete.

This survey will be used for academic/scientific purposes only. Your cooperation is confidential and all answers given are anonymous. You will not be asked for any personal data and you are free to refuse/withdraw from completing the survey at any time.

This survey has been approved by an Ethics Committee with approval code 30/2024

If you have any questions or need further clarification, please contact us by e-mail: jose.pimenta@euvg.pt

* Indica uma pergunta obrigatória

1. I give my consent for the data obtained in this survey to be used for scientific purposes. *

Marcar apenas uma oval.

- ◯ Yes *Avançar para a pergunta 2*

Avançar para a pergunta 2

SECTION 1: VETERINARIANS PROFILE

2. Gender *

Marcar apenas uma oval.

- Male
- Female

3. Academic degree in veterinary medicine *

Marcar apenas uma oval.

- Ph.D
- Master
- Pre-bologna bachelor's degree

4. Do you only practice equine medicine? *

Marcar apenas uma oval.

- Yes
- No (equines and livestock)
- No (equines and small animals)
- No (equines, livestock and small animals)

5. How many years of experience do you have as an equine clinician? *

Marcar apenas uma oval.

- <1 year
- 2-5 years
- 6-10 years
- 11-15 years
- 16-20 years
- >20 years

6. What is your main clinical area of intervention? *

Marcar tudo o que for aplicável.

- [ ] Sports medicine
- [ ] Surgery
- [ ] Reproduction
- [ ] General practice
- [ ] Dentistry
- [ ] Reabilitation
- [ ] Outra: ____________________

7. What is your type of practice *

Marcar apenas uma oval.

- ( ) Ambulatory practice
- ( ) Hospital-based practice
- ( ) Mix (ambulatory + hospital)

8. Geographical area where you mainly do your clinical practice *

Marcar tudo o que for aplicável.

- [ ] North
- [ ] Center
- [ ] South
- [ ] Azores
- [ ] Madeira
- [ ] Outside country

9. Main breeds of horses you work with *

Marcar tudo o que for aplicável.

- [ ] Lusitano
- [ ] Thoroughbred
- [ ] Warmblood
- [ ] Arabian
- [ ] Crossbred
- [ ] Outra: ______________________

10. Disciplines of the horses you work with *

Marcar tudo o que for aplicável.

- [ ] Dressage
- [ ] Jumping
- [ ] Raid
- [ ] Leisure riding
- [ ] Race
- [ ] Working equitation
- [ ] Eventing
- [ ] Outra: ______________________

SECTION 2: EXPERIENCE WITH MUCOCUTANEOUS TUMORS

11. Indicate the types of tumors you have diagnosed/ manage in your clinical activity *

Marcar tudo o que for aplicável.

- [ ] Squamous cell carcinoma
- [ ] Melanoma
- [ ] Sarcoid
- [ ] Cutaneous lymphoma
- [ ] Mast cell tumor
- [ ] Outra: ______________________

12. On average, how many clinical cases of skin tumors do you diagnose/manage per year? *

Marcar apenas uma oval.

- <2
- 3-6
- 7-10
- 11-15
- 16-20
- >20

13. On average, how many clinical cases of sarcoids do you diagnose/manage per year?

Marcar apenas uma oval.

- <2
- 3-6
- 7-10
- 11-15
- 16-20
- >20

14. What is your degree of concern regarding a horse with sarcoids, during a routine clinical examination? *

Marcar apenas uma oval.

- Very high
- High
- Moderate
- Low
- Very low

15. What is your degree of concern regarding a horse with skin sarcoids, during a pre-purchase exam? *

Marcar apenas uma oval.

- Very high
- High
- Moderate
- Low
- Very low

16. Do you alert owners and recommend treatment when you detect a suspecting sarcoid during a clinical examination conducted for an unrelated primary reason? *

Marcar apenas uma oval.

- Yes
- No

17. If you answered "No", explain why:

18. In the case of a lesion suspected of being sarcoid, do you recommend histopathological analysis? *

Marcar apenas uma oval.

- Yes
- No

19. If you answered "No", explain why:

20. Which treatments do you use most often for sarcoids? *

Marcar tudo o que for aplicável.

- [ ] I don't treat sarcoids
- [ ] Classic surgery
- [ ] Laser/electrocautery surgery
- [ ] Elastrator/ligature
- [ ] Acyclovir
- [ ] Intratumoral chemotherapy
- [ ] Intratumoral chemotherapy + surgery
- [ ] Topical AW5 ("Liverpool cream")
- [ ] Electrochemotherapy
- [ ] Immunotherapy (intratumoral BCG)
- [ ] Cryosurgery (liquid nitrogen or other)
- [ ] Radiotherapy
- [ ] Imiquimod
- [ ] Outra: __________

21. Recommends intervention on sarcoids: *

Marcar apenas uma oval.

- ( ) As soon as possible
- ( ) As late as possible
- ( ) Only when they already affect the horses' health/well-being
- ( ) I don't recommend intervening
- ( ) Outra: __________

22. Have you ever referred sarcoid cases? *

Marcar apenas uma oval.

- Yes
- No

23. If you answered "Yes", explain why

24. Do you think it's possible to prevent the appearance of sarcoids in horses? *

Marcar apenas uma oval.

- Yes
- No

25. If you answered "Yes", explain how

26. On average, how many clinical cases of melanomas do you diagnose/manage per year?

Marcar apenas uma oval.

- <2
- 3-6
- 7-10
- 11-15
- 16-20
- >20

27. What is your degree of concern regarding a horse with melanomas, during a routine clinical examination? *

Marcar apenas uma oval.

- Very high
- High
- Moderate
- Low
- Very low

28. What is your degree of concern regarding a horse with melanomas, during a pre-purchase examination? *

Marcar apenas uma oval.

- Very high
- High
- Moderate
- Low
- Very low

29. Do you alert owners and recommend treatment when you detect a suspecting melanoma during a clinical examination conducted for an unrelated primary reason? *

Marcar apenas uma oval.

- Yes
- No

30. If you answered "No", explain why

31. When you're called in to assess a horse with melanomas, it's usually: *

Marcar apenas uma oval.

- It's a tumor that was detected recently, having appeared only a short time ago
- It's an old tumor that has grown large and has become a cause for concern for the owner
- It's an ulcerated tumor
- Outra: ______

32. In the case of a lesion suspected of being melanoma, do you recommend histopathological analysis? *

Marcar apenas uma oval.

- Yes
- No

33. If you answered "No", explain why

34. Which treatments do you use most often for melanomas? *

Marcar tudo o que for aplicável.

- [ ] I don't treat melanomas
- [ ] Classic surgery
- [ ] Laser/electrocautery surgery
- [ ] Intratumoral chemotherapy
- [ ] Intratumoral chemotherapy + surgery
- [ ] Cimetidine
- [ ] Electrochemotherapy
- [ ] Immunotherapy (Oncept® Vaccine)
- [ ] Cryosurgery (liquid nitrogen or other)
- [ ] Radiotherapy
- [ ] Imiquimod
- [ ] Outra: ____________

35. Did you get good results in the melanoma cases in which you used the Oncept ® vaccine? *

Marcar apenas uma oval.

- Yes
- No
- I never used

36. Did you get good results in the melanoma cases in which you used oral cimetidine? *

Marcar apenas uma oval.

- Yes
- No
- I never used

37. Recommends intervention on melanomas: *

Marcar apenas uma oval.

- As soon as possible
- As late as possible
- Only when they already affect the horses' health/well-being
- I don't recommend intervening
- Outra: _______________

38. Have you ever referred cases of melanoma? *

Marcar apenas uma oval.

- Yes
- No

39. If you answered "Yes", explain why

____

40. Have you ever diagnosed metastases (ante or post mortem) in a horse with melanomas? *

Marcar apenas uma oval.

- Yes
- No

41. If you answered "Yes", did the horse(s) show clinical signs compatible with metastasization?

Marcar apenas uma oval.

- Yes
- No

42. If you answered "Yes", please indicate which clinical signs:

43. Have you ever had to euthanize a horse due to the presence of melanomas? *

Marcar apenas uma oval.

- Yes
- No

44. If you answered "Yes", explain why

45. Do you think it's possible to prevent the appearance of melanomas in horses? *

Marcar apenas uma oval.

- Yes
- No

46. If you answered "Yes", explain how

47. On average, how many clinical cases of squamous cell carcinoma do you diagnose/manage per year?

Marcar apenas uma oval.

- <2
- 3-6
- 7-10
- 11-15
- 16-20
- >20

48. What is your degree of concern regarding a horse with squamous cell carcinoma, during a routine clinical examination? *

Marcar apenas uma oval.

- Very high
- High
- Moderate
- Low
- Very low

49. What is your degree of concern regarding a horse with squamous cell carcinoma, during a pre-purchase examination? *

Marcar apenas uma oval.

- Very high
- High
- Moderate
- Low
- Very low

50. Do you alert owners and recommend treatment when you detect a suspecting squamous cell carcinoma during a clinical examination conducted for an unrelated primary reason? *

Marcar apenas uma oval.

- Yes
- No

51. If you answered "No", explain why:

52. In the case of a lesion suspected of being squamous cell carcinoma, do you recommend histopathological analysis? *

Marcar apenas uma oval.

- Yes
- No

53. If you answered "No", explain why

54. Which treatments do you use most often for squamous cell carcinomas? *

Marcar tudo o que for aplicável.

- I don't treat squamous cell carcinomas
- Classic surgery
- Laser/electrocautery surgery
- Cryosurgery (liquid nitrogen or other)
- Intratumoral chemotherapy
- Intratumoral chemotherapy + surgery
- COX-2 inhibitors
- Electrochemotherapy
- Radiotherapy
- Outra: ____

55. Recommends intervention on squamous cell carcinomas: *

Marcar apenas uma oval.

- As soon as possible
- As late as possible
- Only when they already affect the horses' health/well-being
- Opção 4I don't recommend intervening
- Outra: ______________________

56. Have you ever referred cases of squamous cell carcinoma? *

Marcar apenas uma oval.

- Yes
- No

57. If you answered "Yes", explain why

____

58. Do you think it is possible to prevent the appearance of squamous cell carcinomas in horses? *

Marcar apenas uma oval.

- Yes
- No

59. If you answered "Yes", explain how

____
____
____
____
____

SECTION 3: VETERINARIANS OPINION ON OWNERS AWARENESS ABOUT MUCOCUTANEOUS TUMORS

60. How would you rate the owners' level of concern regarding the presence of a sarcoid during a routine clinical examination? *

Marcar apenas uma oval.

- Very high
- High
- Moderate
- Low
- Very low

61. How would you rate the buyers' level of concern regarding the presence of a sarcoid during a pre-purchase examination? *

Marcar apenas uma oval.

- Very high
- High
- Moderate
- Low
- Very low

62. Do you feel that the owners are interested and willing to invest in treatment for sarcoids?

Marcar apenas uma oval.

- Yes
- No

63. How would you rate the owners' level of concern regarding the presence of a melanoma during a routine clinical examination? *

Marcar apenas uma oval.

- Very high
- High
- Moderate
- Low
- Very low

64. How would you rate the buyers' level of concern regarding the presence of a melanoma during a pre-purchase examination? *

Marcar apenas uma oval.

- Very high
- High
- Moderate
- Low
- Very low

65. Do you feel that the owners are interested and willing to invest in treatment for melanomas? *

Marcar apenas uma oval.

- Yes
- No

66. How would you rate the owners' level of concern regarding the presence of a squamus cell carcinoma during a routine clinical examination? *

Marcar apenas uma oval.

- Very high
- High
- Moderate
- Low
- Very low

67. How would you rate the buyers' level of concern regarding the presence of a squamous cell carcinoma during a pre-purchase examination? *

Marcar apenas uma oval.

- Very high
- High
- Moderate
- Low
- Very low

68. Do you feel that the owners are interested and willing to invest in treatment for squamous cell carcinoma? *

Marcar apenas uma oval.

- Yes
- No

Este conteúdo não foi criado nem aprovado pela Google.

Google Formulários
